# A Pan-Cancer Landscape of HOX-Related lncRNAs and Their Association With Prognosis and Tumor Microenvironment

**DOI:** 10.3389/fmolb.2021.767856

**Published:** 2021-11-05

**Authors:** Wei Shao, Qian Ding, Yugang Guo, Juan Xing, Zheng Huo, Zhan Wang, Qian Xu, Yue Guo

**Affiliations:** ^1^ Henan Provincial Engineering Laboratory of Insects Bio-Reactor, Nanyang Normal University, Nanyang, China; ^2^ Henan Provincial Nanyang Central Hospital, Nanyang, China

**Keywords:** homology cassette HOX, long non-coding RNA, prognostic signature, cancer immunity, immune infiltration

## Abstract

The highly conserved homology cassette family (HOX) as well as 18 referenced long non-coding antisense transcripts (HOXATs) play vital roles in the development of some cancers. Nevertheless, their expression patterns as well as their association with cancer prognosis and the tumor microenvironment (TME) in pan-cancers are still unclear. Here, based on public databases, the expression levels of HOXATs, their prognostic potentials, and correlation with tumor mutation burden (TMB), immune cell infiltration, immune subtype, immune response-related genes, and stemness scores corresponding to 33 tumor types were analyzed systematically using R language. The results of the analysis indicated that different cancer tissues show different HOXAT expression profiles. Further, HOXAT expression showed association with cancer prognosis and immune and stemness regulation. Gene set enrichment analysis also demonstrated that HOXATs participate in cancer- and immune-related pathways, and based on their expression levels, HOTAIRM1 and HOXB-AS1 showed potential involvement in oncogenesis as well as possible involvement in immune regulation across a variety of cancer types. Further investigation also confirmed a significantly higher expression of HOXB-AS1 in GBM than in lower grade glioma tissues. Importantly, *in vitro* cell function experiments indicated that HOXB-AS1 supports cancer stem cell and plays a fundamental role in glioma metastasis. In conclusion, our results provide valuable resources that can guide the investigation of the mechanisms related to the role of HOXATs in cancers as well as therapeutic analysis in this regard.

## Introduction

For Long non-coding RNAs (lncRNAs), as one of the key regulators of cancer, function not only as important molecular markers for cancer prognosis, but also as markers of molecular characteristics at the pan-cancer level. Increasing studies have shown that several lncRNAs affect cancer tumorigenesis and contribute to cancer pathogenesis (Akbari et al., 2020; Bi et al., 2020).

The tumor microenvironment (TME), which contains multiple types of stromal cells, tumor cells, cancer stem cells (CSCs), tumor-associated immune cells and others, plays a major role in tumor growth and metastasis, and particularly, the immune microenvironment constitutes a vital element of tumor biology. Immune system dysregulation can be a major cause of cancer development (Chen et al., 2019a; Carlsten and Järås, 2019). The most recent studies suggest that CSCs represent another important component of the TME that have differentiation abilities to generate the original lineage cells for regulating tumor occurrence, development, recurrence, metastasis and drug resistance. It has also been suggested that the activities of CSCs and immune cells represent the potential mechanisms that link the TME and cancer. Recently, increasing evidence has indicated that lncRNAs play fundamental roles in the regulation of the immune microenvironment and are closely associated with the development and progression of various CSCs. For example, the lncRNA, NeST has been shown to be related to T-cell activation, and is critical for immune response regulation (Chen et al., 2019b), while the lncRNA, NRON has been shown to maintain a resting state of T cells by sequestering phosphorylated NFAT in the cytoplasm (Chen et al., 2020). Moreover, it has also been demonstrated that the lncRNA, lnc-sox5, modulates CRC tumorigenesis by unbalancing TME (Chen et al., 2016), while the lncRNA, LINK-A functions as an oncogene that promotes tumor immune evasion by sensitizing T cells (Dong et al., 2018). Additionally, it has been observed that the depletion of oncogenic HotairM1 promotes the self-renewal of CSCs *via* the HOXA1-Nanog signaling loop. It has also been observed that lncTCF7 is required for liver CSC self-renewal and tumor propagation (Chi et al., 2019). However, further studies on lncRNAs and their roles in the TME of different cancer types still require investigation.

Transcription factors are encoded by homeobox (HOX) genes, which are assigned to a group of 13 orthologs and paralogs in four clusters and share similar positions in the homozygous frames, with 9–11 genes per cluster (Cruz et al., 2017). Multiple reports have shown that the dysregulation of 39 HOX genes is implicated in various aspects of cancer. Similar to protein-coding transcripts, accumulating experimental evidence indicates that lncRNA transcripts in the Hox gene locus, with 18 referenced non-coding antisense transcripts (HOXATs) are implicated in cancer progression. These 18 HOXATs are located in four HOXATs clusters, with the HOXA region consisting of HOTAIRM1, HOXA-AS2, HOXA-AS3, HOXA10-AS, HOXA11-AS, and HOTTIP, the HOXB region consisting of HOXB-AS1, HOXB-AS2, HOXB-AS3, HOXB-AS4, and PRAC2, the HOXC region consisting of HOXC13-AS, HOXC-AS1, HOXC-AS2, HOXC-AS3, and HOTAIR, and the HOXD region consisting of HAGLR and HOXD-AS2. For example, it has been reported that the expression levels of HOXA10-AS, HOXA11-AS, and HOXA-AS are significantly upregulated in lung adenocarcinoma (LUAD), and their high expression levels in such a cancer type are associated with shorter survival periods in patients (Hao and Yu, 2018; Dasti et al., 2020). Further, it has also been observed that the expression levels of HOTAIRM1, HOXA10-AS, HOXA10-AS, HOXA11-AS, HOXA-AS2, HOXA-AS3, HOXB-AS1, HOXC13-AS, HOXC-AS3, and HOXD-AS2 are upregulated in glioma, and are positively correlated with malignancy (Li et al., 2017; Li et al., 2020a), and importantly, some studies have shown that HOXATs can play fundamental roles in immune system regulation (Li et al., 2020b; Liang et al., 2020). However, the different expression levels and the specific functions of the individual HOXATs in pan-cancer as well as their association with the TME still remain unclear. Therefore, an in-depth investigation and analysis of their potential roles in pan-cancer is warranted.

Hence, in this study, we attempted to explore and analyze the expression profile and prognostic significance of HOXATs across multiple cancer types and investigated the potential role of the HOXATs in the modulation of the TME as well as stemness score. Additionally, we also explored and verified the potential biological function and pathways of the HOXATs with respect to the different aspects of cancer.

## Materials and Methods

### Literature Review and meta-Analysis

To identify all the publications related to HOXATs in patients with cancer, the PubMed database and Web of Science were searched for literature using the following terms, which correspond to 16 out of the 18 referenced non-coding antisense transcripts: HOXAIRM1, HOXA-AS3, HOXA10-AS, HOXA11-AS, HOTTIP, HOXB-AS1, HOXB-AS2, HOXB-AS3, HOXB-AS4, HOXC13-AS, HOXC-AS1, HOXC-AS2, HOXC-AS3, HOTAIR, HAGLR, and HOXD-AS2. The exclusion criteria were as follows: 1) repeated studies and 2) reviews, comments, letters, case reports, and conference articles. Further, subsequent meta-analysis was performed for HOTTIP and HAGLR using comprehensive Meta‐Analysis V315.

### Datasets Used

In this study, datasets from the public TCGA pan-cancer data portal (https://xenabrowser.net/datapages/), containing 10,635 samples spanning 33 cancer types with clinically annotated multi-omic data (clinical data, stemness scores based on mRNA (RNAss) and DNA methylation (DNAss), and immune subtypes) were used for analyses.

### Correlations Between HOXATs and Tumor Microenvironment

ESTIMATE-based immune and stromal scores were used to analyze the infiltration levels of immune and stromal cells in the different tumor types. Six immune subtypes were defined to measure the levels of immune infiltrate types in the TME based on the TCGA pan-cancer database. Further, tumor stemness features extracted from the transcriptomics and epigenetics of TCGA tumor samples were used to measure the stem-cell-like features of the tumor cells. CIBERSORT was then used for the calculation of the TMB scores of the tumor cells and the correlation between the TMB and HOXATs levels was analyzed (Lin et al., 2020). Further, the Spearman or Pearson correlation coefficients obtained were then used to evaluate the relationships between the expression levels of the HOXATs and RNAss, DNAss, immune score, estimate score, TMB, immune infiltration, and the expression of immune checkpoint genes as well as tumor stemness markers.

### Gene Set Enrichment Analysis

The GSEA was performed using the Java v3.0 desktop application (http://software.broadinstitute.org/gsea/index.jsp).

### Cells Culture

Two human glioma cell line samples (U251 and HS683) were obtained from Cell Culture Center, Chinese Academy of Medical Sciences (Shanghai, China). The cells were maintained in a medium containing Dulbecco’s Modified Eagles Medium (DMEM), 10% fetal bovine serum (Invitrogen, Carlsbad, CA, United States), and 1% penicillin–streptomycin solution. Thereafter, they were cultured under standard cell culture conditions consisting of 5% CO_2_ and 95% relative humidity at 37 C.

### lncRNA Knockdown

U251 and HS683 cells in the logarithmic growth phase were incoculated into 6-well plates, after the cells grew to 50–60% fusion. The lncRNA-HOXB-AS1 smart silencer (a mixture containing three siRNAs and three antisense oligonucleotides) and its negative control, lncRNA smart silencer, NC, synthesized by RiboBio were used (Guangzhou, China). The cells were transfected using Lipofectamine RNAiMAX (Invitrogen, Carlsbad, CA, United States) following the manufacturer’s protocol.

### Sphere Formation Assay

Single human glioma cell line suspensions were washed twice with serum-free phosphate buffer solution (PBS) and transferred into a serum-free stem cell complete medium (containing 20 μg/L bFGF and 20 μg/L EGF) followed by the addition of B27. Thereafter, the cells were cultured at 37 C in 5% CO_2_, and sphere formation was analyzed.

### Invasion Assay and Wound Healing Assay

To realize invasion assay, transwell chambers (Corning, Tewksbury, MA, United States) were coated with Matrigel (dilution 1:8; BD Biosciences, Bedford, MA, United States). Thereafter, a 120-μL serum-free medium containing 5 × 10^4^ cells was added to the upper chamber, while a 600-μL complete medium containing 10% FBS was added to the lower chamber. After transfaction, the cells on the lower surface were fixed with methanol and stained with 0.1% crystal violet and then captured using an inverted microscope (AMG, United States). To realize the wound healing assay, U251 and HS683 cells were incoculated into 6-well plates. When the cell cultures reached 80% confluence, the cells were scraped using a 10-μL micropipette tip. Thereafter, images were captured using an inverted microscope with a camera (AMG, United States). The distance travelled by the migrating cells was quantified to assess cell migration capacity using ImageJ software version 1.46 (National Institutes of Health, Bethesda, MD, United States).

### MTT Assay

Cell viability was monitored using an MTT cell viability assay kit (Sigma-Aldrich, St. Louis, MO, United States). The transfected cells were seeded in 96‐well plates. Thereafter, 10 μL MTT (5 mg/ml) was added to each well and incubation was continued for 2 h, followed by the dissolution of the precipitate formed in 100 μL of dimethyl sulfoxide (DMSO). Absorbance measurements were then performed at 570 nm under a microplate spectrophotometer.

### Bioinformatics and Statistical Analyses

All computational and statistical analyses were performed using R software v4.0.3 and Prism 8 GraphPad (https://www.graphpad.com/scientific-software/prism/). Correlations between stemness markers and HOXAT expression levels were analyzed using the GEPIA online dataset (http://gepia.cancer-pku.cn/), which includes the gene expression profiles of different cancers and normal tissues in the TCGA database as well as genotype-tissue expression (GTEx). *p*-values < 0.05 were considered statistically significant. Further, the results were adjusted for multiple comparisons.

## Results

### Brief Literature Review

We analyzed the connection between the expression of HOXATs and prognosis in patients with cancer by exploring all published related literature. A total of 1,802 potential studies were identified, and after title and abstract screening, 65 studies were found to be eligible for inclusion. Next, we examined the full text of these 65 articles, in which most of the relationship between HOXATs and their related genes, as well as the mechanisms of regulation were investigated. Conversely, we observed that studies in which the relationship between the HOXATs and the immune microenvironment in pan-cancers were investigated were scarce. Further, no study aimed at investigating the immune response of HOXATs in tumors was identified (Table 1).

**TABLE 1:** A brief review of the literature. The related interactive genes of HOXATs and involvement in functional role in different cancers. N (T/N):number (tumor/normal). ADC: Lung adenocarcinoma; AF: Atrial fibrillation; BRCA: Breast invasive carcinoma; COAD: Colon adenocarcinoma; CRC: colorectal carcinoma; EC: esophageal carcinoma; EC: endometrial cancer; EO: epithelial ovarian cancer; HCC: hepatocellular carcinoma; HNSCC: Head and neck squamous cell carcinoma; GC: gastric carcinom; GBM: Glioblastoma multiforme; IMA: Invasive mucinous adenocarcinoma of the lung; LGG: Brain Lower Grade Glioma; LUAD: lung adenocarcinoma; MM: Multiple myeloma; NSCL: Cnonsmall-cell lung cancer; NPC: Nasopharyngeal carcinoma. NSCL: Cnonsmall-cell lung cancer; NPC: Nasopharyngeal carcinoma. OS: osteosarcoma; OSCC: Oral squamous cell carcinoma; OC: oral carcinoma; OC: oral carcinoma; PAAD: Pancreatic adenocarcinoma; PDAC: Pancreatic ductal; PTC: papillary thyroid cancer; SCLC: small cell lung cancer; UM: uveal melanoma; UCEC: Uterine Corpus Endometrial Carcinoma.

Gene	Year	County	Cancer types	N (T/N)	Related axis and genes
HOTAIRM1	2020	United States	ccRCC	mES KH2 cell line	Hypoxia-Inducible Factor 1
	2020	China	ADC	92 (46/46)	miR-498/WWOX axis
	2019	China	EC	100 (50/50)	HOXA1/Wnt
	2018	China	GBM	90 (70/20)	HOXA1/TSS
	2018	China	HCC	60 (30/30)	Wnt
HOXA-AS2	2020	China	PCa	172 (86/86)	miR-509-3p/PBX3
	2020	China	UCEC	75 (35/30)	MiRNA-302c-3p/ZFX/YKL-40
	2019	China	OS	54 (27/27)	E2F3/miR-124-3p
	2019	China	PTC	68 (34/34)	miR-15a-5p/HOXA3
	2019	China	NSCLC	80 (40/40)	microRNA-216a-5p
	2019	China	GBM	100 (50/50)	EZH2/LSD1
	2018	China	HCC	58 (29/29)	miR-520c-3p/GPC3
	2017	China	PAAD	56 (28/28)	EZH2/LSD1
	2017	China	CRC	120 (60/60)	HOXA-AS2C/EMT
	2017	China	BRCA	76 (32/32)	miR-520c-3p
	2015	China	GC	110 (55/55)	PRC2/P21/PLK3/DDIT3
HOXA-AS3	2021	China	GC	164 (82/82)	miR-29a-3p/LTβR/NF-κB regulatory
	2020	China	GBM	507 (502/5)	miR-455-5p/USP3
	2019	China	HCC	152 (76/76)	miR-29c/BMP1/MEK/ERK
	2018	China	LUAD	18 (9/9)	HOXA6/NF110
HOXA10-AS	2018	China	GBM/LGG	79 (59/20)	HOXA10
	2018	China	LAD	86 (43/43)	ELK1/Wnt/β-catenin
	2015	United States	EOC	3210 (1201/2009)	rs17427875
HOXA11-AS	2020	China	OSCC	100 (50/50)	miR-98-5p/YBX2
	2019	China	GBM	86 (43/43)	miR-130a-5p/HMGB2
	2019	China	OS	122 (61/61)	miR-125a-5/Rab3D
	2018	China	LUAD	535 (267/268)	miR-454-3p/Stat3
	2017	China	CRC	30 (15/15)	miR-125a-5p/PADI2
	2017	China	BRCA	136 (68/68)	MDA-MB-231/MCF-7
	2017	China	NSCLC	78 (38/39)	miR-200b/EZH2/DNMT1
HOTTIP	2020	China	BRCA	unknow	miR-148a-3p/Wnt/β‐catenin
	2019	China	GC	58 (30/28)	miR-218/HMGA1
	2019	China	OC	78 (53/25)	HOXA11/IL-6/PD-L1
	2017	China	GBM	116 (60/56)	HIF-1/HOTTIP/miR-101/ZEB
	2017	China	PDAC	180 (90/90)	WDR5/HOXA9/Wnt
HOXB-AS1	2021	China	GBM	unknow	HOXB-AS1-ILF3-HOXB2/HOXB3
	2020	China	MM	120 (60/60)	ELAVL1/FUT4
	2020	China	GBM/EC	128 (64/64)	miR-149-3p/Wnt10b/Wnt/β-catenin/mir-149-3p
	2019	China	LGG/GBM	640 (486/154)	miR-885-3p/HOXB2
HOXB-AS2	2020	China	AF	unknow	HOTAIRM1/HOXA-AS2/TNF
HOXB-AS3	2019	China	EOC	unknow	HOXB/Wnt/catenin
	2017	China	CRC	180 (90/90)	hnRNP A1
HOXB-AS4	2019	Japan	PAAD	31 (26/4)	SIM1/MIR129-2/NR1I2
PRAC2	2019	Canada	BRCA	2136 (1992/144)	GRCh37/hg19
HOXC13-AS	2020	China	OSCC	300 (150/150)	miR-378g/HOXC13
	2020	China	HNSCC	185 (141/44)	LINC00958
	2020	United States	CRC	78 (39/39)	HOTAIR/HOXC-AS3/HOXC10/HOXC13
	2019	China	GBM	27 (20/7)	miR-122-5p
	2019	China	HCC	394 (197/197)	miR497-5p/PTEN
HOXC-AS1	2020	Japan	CRPC	14 (8/6)	PRKAG2-AS1/CRPC-Lnc/mitochondria
	2019	China	GC	70 (35/35)	miR-590-3p/MYC
HOXC-AS2	2020	China	GC	407 (375/32)	HOXC8/HOXC9/HOXC10/HOXC11/HOXC12/HOXC13
	2019	China	GBM	9 (6/3)	HOXB13
HOXC-AS3	2020	China	IMA	unknow	FOXM1/FUS/FOXM1/IMA
	2018	China	GC	112 (56/56)	YBX1
HOTAIR	2020	China	NSCLC	104 (62/42)	EGFR-TKIs
	2019	China	HCC	424 (374/50)	RAB35/VAMP3 SNAP23
	2019	China	CRC	84 (42/42)	miR-214/ST6GAL1
	2018	China	BRCA	40 (20/20)	miR‐20a‐5p/HMGA2
	2018	United States	GBM	106 (66/40)	HOTAIR-205/Bromodomain Containing 4 protein
HAGLR	2020	China	COAD	50 (25/25)	miR-185-5p/CDK4/CDK6
	2019	China	EC	118 (73/45)	microRNA-143-5p/LAMP3
HOXD-AS2	2020	China	GC	158 (79/79)	HOXD8/PI3K/Akt
	2018	India	GBM	28 (19/9)	INK4b/ARF/INK4a

### HOXATs Expression and Correlation With Pan-Cancers

**FIGURE 1:**
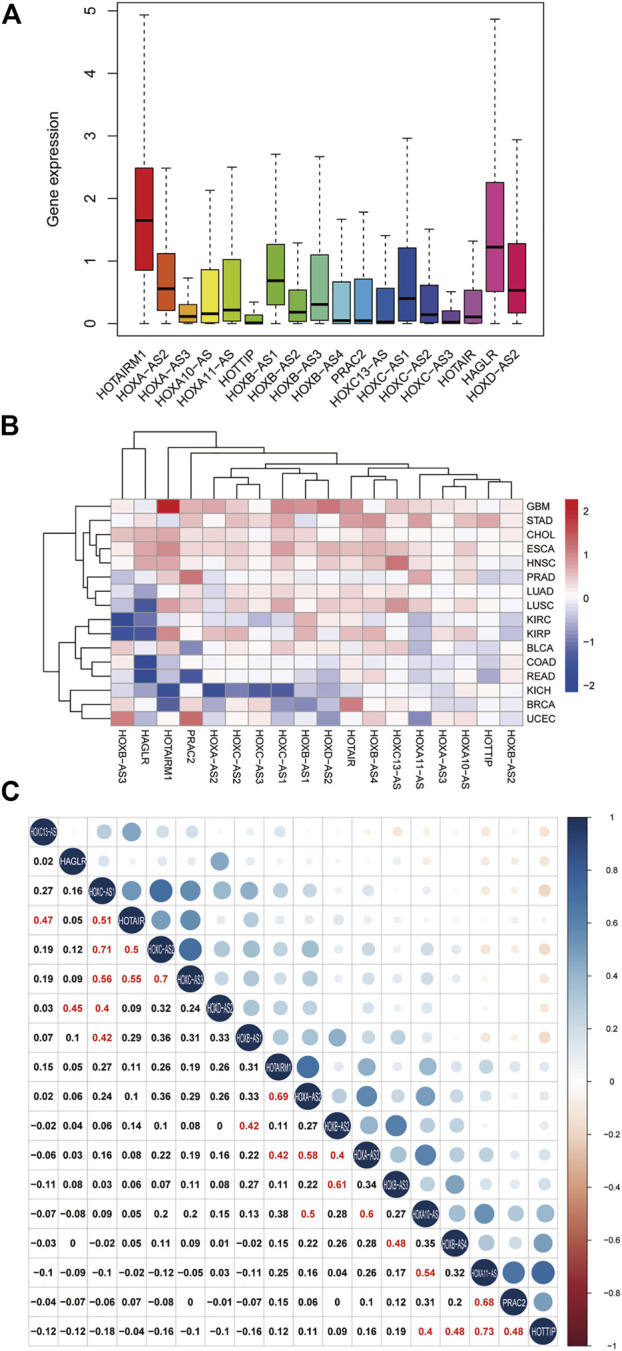
Homeobox antisense transcripts (HOXATs) are abnormally expressed in human pan-cancer. Boxplot showing the distribution of the HOXATs expression across 33 cancer types **(A)**. Heatmap showing the difference in the HOXATs expression between the primary tumor and the adjacent normal tissues, which was based on log2 (fold change) of 16 cancer types that had more than five adjacent normal samples **(B)**. Correlation plot based on Spearman’s correlation test showing the correlation of gene expression among the 18 HOX-related long non-coding RNAs (lncRNAs) across the 33 cancer types **(C)**.

To clarify the intrinsic expression pattern of HOXATs, we described the differential expression of HOXATs considering the 33 available cancer types in the TCGA pan-cancer database. Notably, most of the HOXATs showed a striking intra- and inter-tumor heterogeneity in these different cancer types. Among them, HOTAIRM1 and HAGLR showed relatively higher expression levels in all the cancer types than the other HOXATs (Figure 1A; Supplementary Figures S1–S18). Further, we systematically investigated the expression levels of all 18 genes in primary patient tumor samples corresponding 16 cancer types. This investigation also included at least five normal samples. For instance, the expression levels of HOTAIRM1 showed significant inter-tumor heterogeneity in esophageal carcinoma (ESCA), GBM, and kidney papillary cell carcinoma (KIRP), which are associated with very high HOTAIRM1 expression levels. Conversely, breast cancer (BRCA), colon adenocarcinoma (COAD), kidney chromophobe (KICH), and rectum adenocarcinoma (READ) were found to be characterized by lower HOTAIRM1 expression levels (*p* < 0.05; Figure 1B). Therefore, these results indicated the existence of intrinsic differences between the different tumor types with respect to the expression of HOXATs.

Next, we explored the potential relationship between the HOXATs. In this regard, Pearson correlation analysis revealed the following significant correlations between the HOXATs: HOTAIRM1 with HOXA-AS2 and HOXA-AS3; HOXA-AS2 with HOXA-AS3 and HOXA10-AS; HOXA-AS3 with HOXA10-AS and HOXB-AS2; HOXA10-AS with HOXA11-AS and HOTTIP; HOXA11-AS with PRAC2; HOTTIP with HOXB-AS4 and PRAC2; HOXB-AS1 with HOXB-AS2 and HOXC-AS1; HOXB-AS2 with HOXB-AS3; HOXB-AS3 with HOXB-AS4; HOXC13-AS with HOTAIR; HOXC-AS1 with HOXC-AS2, HOXC-AS3, and HOTAIR; HOXC-AS2 with HOXC-AS3 and HOTAIR; HOXC-AS3 with HOTAIR; and HAGLR with HOXD-AS2 (R > 0.4; Figure 1C). These significant correlations indicated that several HOXATs interact with each other during the pathogenesis of pan-cancer. Interestingly, HOTAIRM1 and HOXA-AS2 showed positive correlation with the other 16 HOXATs, suggesting that they possibly exert a regulatory effect on all the other 16 HOXATs and may be critical factors in cancer development. Notably, the correlation coefficients between HOXA11-AS and HOTTIP, HOXC-AS1 and HOXC-AS2 as well as that between HOXC-AS2 and HOXC-AS3 were all ≥ 0.7, suggesting that they possibly share common features and functions.

### Prognostic Analysis of HOXATs in Pan-Cancer

**FIGURE 2:**
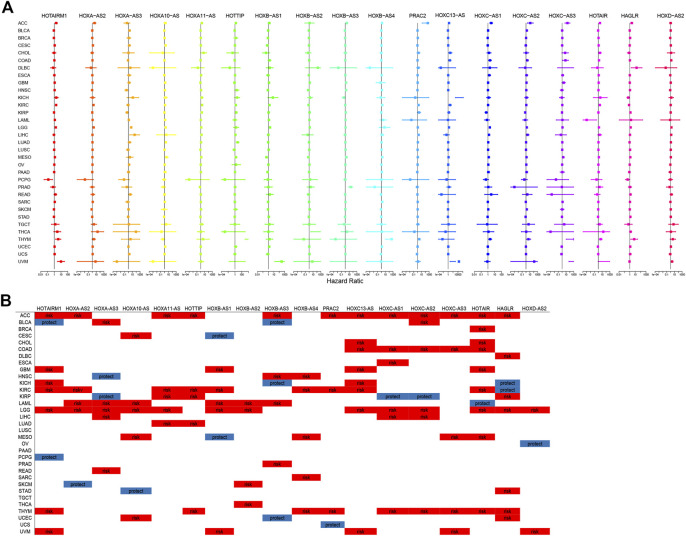
Correlation between the homeobox antisense transcripts (HOXATs) expression and the overall survival (OS). Relationship between the HOXATs gene expression and the OS in patients with different cancer types. Forest plots of the hazard risk (HR) with 95% confidence intervals in the OS across different cancer types showed an advantage (HR < 1) and a disadvantage (HR > 1) in survival with increasing HOXATs expression **(A, B)**. Statistical significance was set at *p* < 0.05.

The prognostic significance of HOXATs in the different cancer types was analyzed using Cox regression (Figures 2A,B). In cancers, including adenoid cystic carcinoma (ACC), KIRC, LGG, and thymoma (THYM), approximately half or more than half of the HOXATs showed association with worse survival of patients. Additionally, it was also observed that certain HOXATs possibly exerted obviously different prognostic effects across the various cancer types. For instance, HOXA11-AS predicted poor prognosis in patients with ACC, KICH, KIRC, LGG, and lung adenocarcinoma (LUAD), HOTTIP predicted poor prognosis in patients with ACC, KICH, KIRC, LGG, LUAD, and uveal melanoma (UVM), and HOTAIRM1 predicted poor prognosis in patients with ACC, GBM, KICH, KIRC, LGG, THYM, and UVM patients, but was found to be associated with a survival advantage for bladder urothelial carcinoma (BLCA) and pheochromocytoma/paraganglioma (PCPG). Further, HOXC-AS1 predicted poor prognosis in patients with ACC, COAD, ESCA, acute myeloid leukemia (LAML), LGG, and THYM, but showed association with a survival advantage for KIRP. Furthermore, HOXC- AS2 predicted poor prognosis for ACC, BLCA, COAD, LGG, liver hepatocellular carcinoma (LIHC), THYM, but was associated with a survival advantage for KIRP. It was also observed that HOTAIR predicted poor prognosis for ACC, BRCA, cholangiocarcinoma (CHOL), COAD, GBM, KIRC, LGG, mesothelioma (MESO), and THYM, but was associated with a survival advantage for LAML. Hence, these results indicated that the different expression levels of the HOXATs show different prognostic associations in the different cancer types.

Moreover, this brief review of the literature indicated the existence of an association between HOXATs and OS in different cancer types. To further validate this aforementioned results, meta-analysis involving HOTTIP and HAGLR was also performed. The results obtained indicated that elevated HOTTIP (Liu et al., 2020) and HAGLR (Liu et al., 2019) expression levels could predict poor prognosis in cancer patients, suggesting that HOTTIP and HAGLR might serve as novel prognostic biomarkers for cancer (Supplementary Figure S19). Moreover, given that several HOXATs influenced LGG prognosis, K-M survival analysis for OS and DSS (Disease Free Survival) were performed to further evaluate the prognostic value of the HOXATs in LGG, based on the TCGA database (Supplementary Figures S20,21). The results obtained showed that the overexpression of all 18 HOXATs could predict poor prognosis in patients with LGG, suggesting that HOXATs might serve as novel prognostic biomarkers for LGG.

### Association Between HOXATs and the Immune Microenvironment in Pan-Cancer

**FIGURE 3:**
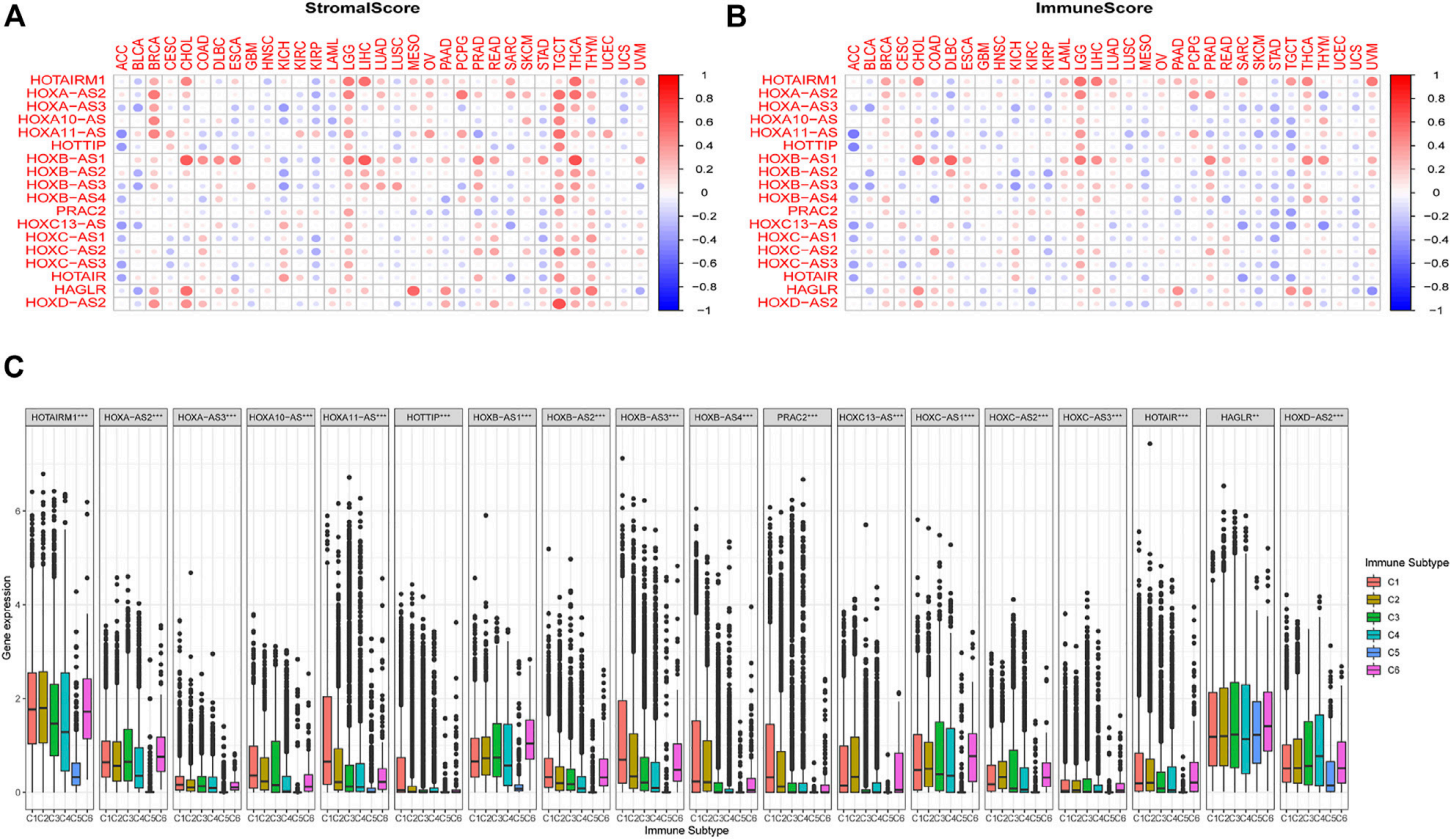
Relationship between the expression of the homeobox antisense transcripts (HOXATs) and the tumor microenvironment. The correlation matrix of the HOXATs expression and the stromal cells **(A)** as well as the immune cells **(B)** of 33 different cancer types based on the estimation algorithm. The Spearman correlation analysis was used to test the immune scores and estimate scores. The size of each point represents the absolute value of the correlation coefficient, where the larger the size, the higher the correlation (the higher the absolute value, the higher the correlation coefficient). Relationship between the expression of the HOXATs and the immune-infiltrating subtypes across all cancer types **(C)**. C1: wound healing, C2: INF-r dominant, C3: inflammatory, C4: lymphocyte depleted, C5: immunologically quiet, and C6: TGFβ dominant.

We further examined the association between the expression levels of HOXATs and the infiltration levels of stromal and immune cells and tumor purity, which was defined based on stromal scores, immune scores, and estimate scores using the ESTIMATE algorithm (Figures 3A,B). The higher stromal and immune scores indicated lower tumor purity, and as expected, the degree of association between the HOXATs and stromal scores for different cancer types varied over a wide range. In cancers, including BRCA, LGG, tenosynovial giant cell tumor (TGCT), thyroid carcinoma (THCA), and THYM, approximately half or more than half of the HOXATs showed positive association with the stromal score of the patients. However, some of the HOXATs showed negative association with stromal scores in patients with ACC, BLCA, and KIRP. Additionally, certain HOXATs displayed different correlations across various cancer types. For instance, HOXB-AS1 (*p* < 0.001) showed the highest correlation with stromal scores across the different cancer types followed by HOTAIRM1 (*p* < 0.001), and HOXA-AS2 (*p* < 0.05). More specifically, we observed a significant positive correlation between HOXB-AS1 and the stromal scores of CHOL, ESCA, LIHC, LGG, and THCA; HOTAIRM1 and the stromal scores of CHOL, LGG, LIHC, and THCA; HOXA-AS2 and the stromal scores of BRCA, TGCT, and THCA, as well as HOXD-AS2 and the stromal score of TGCT (*p* < 0.0001). Conversely, there was a significant negative correlation between HOXA11-AS and the stromal scores of ACC and pancreatic adenocarcinoma (PAAD), and HOTAIR and the stromal scores of ACC and sarcoma (SARC) (*p* < 0.001). Further, we also analyzed the correlation between HOXATs and the immune score. Thus, we observed a significant positive correlation between HOXB-AS1 and the immune scores of CHOL, diffuse large B-cell lymphoma (DLBC), LGG, LIHC, prostate adenocarcinoma (PRAD), and THCA, and between HOTAIRM1 and the immune scores of CHOL, LGG, LIHC, and THCA (Figure 3B).

To further clarify the relationship between each of the HOXATs and the immune components, we determined the correlation between HOXATs and the immune infiltrates in the cancer tumors. Six classes of immune infiltrates corresponding either to tumor promotion or suppression, namely, C1 (wound healing), C2 (INF-r dominant), C3 (inflammatory), C4 (lymphocyte depleted), C5 (immunologically quiet), and C6 (TGFβ dominant), were identified. Patients characterized by C3 and C5 immune subtypes showed significantly better survival rates than those characterized by the other immune subtypes, whereas patients with C4 and C6 subtypes showed the least favorable survival, given that C1 and C2 as well as C4 and C6 showed associated with more aggressive immune subtypes (Li et al., 2019). A comparison of the expression levels of HOXATs corresponding to the different immune infiltrate subtypes showed a strong relationship between the expression of HOXATs genes and the immunity of the patients (*p* < 0.001; Figure 3C). Most of the HOXATs showed a lower expression level in the C5 immune subtype, indicating that they possibly play tumor promoter roles. Conversely, HAGLR and HOXD-AS2 were highly expressed in all the six immune subtypes, showing higher expression in C5, which in future, can be used as a new direction for immunosuppression. Additionally, higher levels of HOXA11-AS, HOTTIP, HOXB-AS3, HOXB-AS4, PRAC2, and HOXC13-AS were found to be associated with types 1, 2, and 6 infiltrates (i.e., C1, C2, and C6), indicating the tumor promoter role of these gene members, given that patients belonging to these categories, who showed a higher cancer cell proliferation rate as well as TGFβ enrichment, had lower survival rates (Lu et al., 2018). Conversely, HOTAIRM1, HOXA-AS2, HOXA10-AS, HOXB-AS1, HOXC-AS1, HOXC-AS2, and HOXD-AS2 showed association with C3 and C6 immune subtypes, and the decreased expression in the C5 subtype indicated that the differential expression of HOXATs in the different immune subtypes may partially explain why HOXATs played contrasting roles in the prognosis of various cancers.

### Correlation Between HOXAT Expression and Immune Marker Sets

**FIGURE 4:**
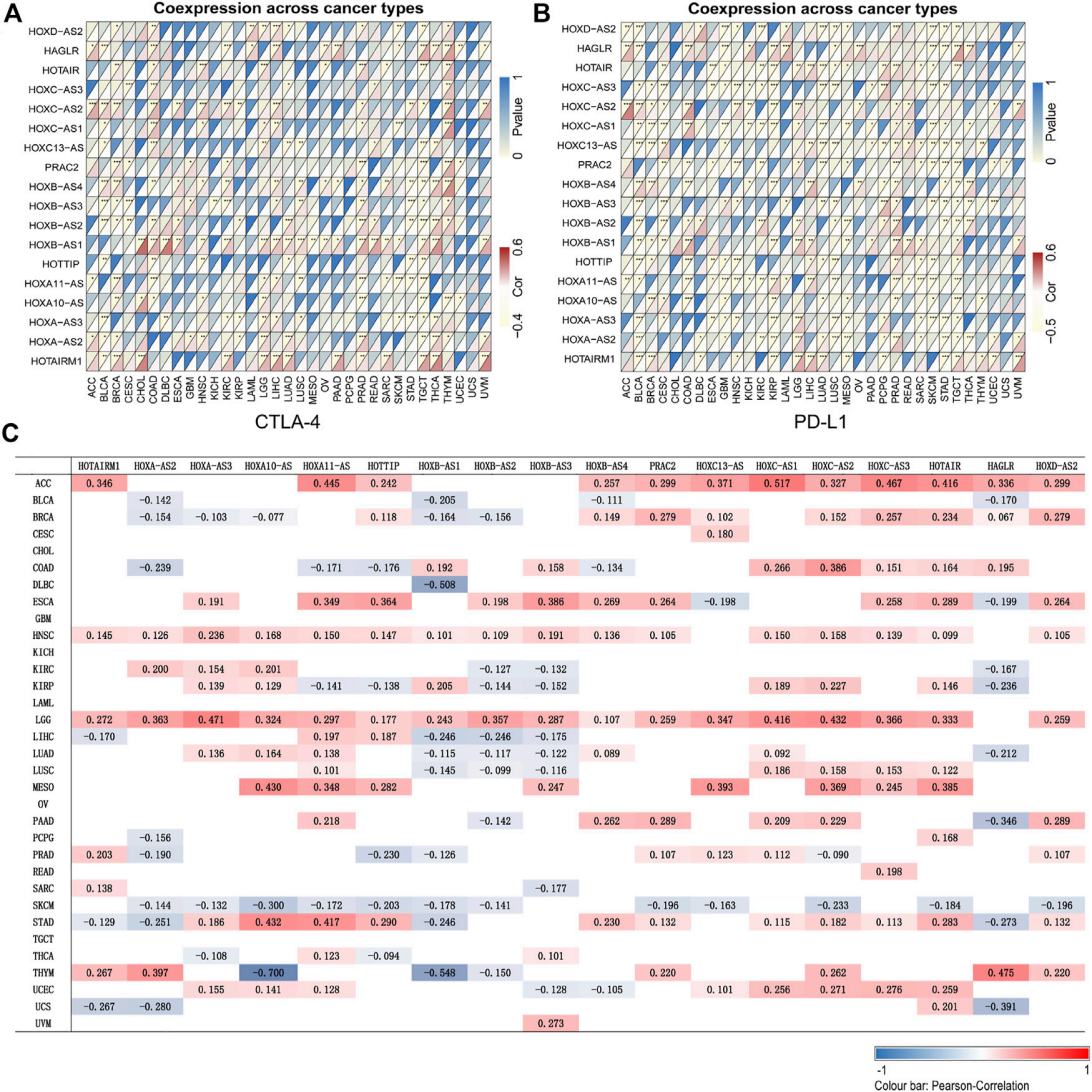
Association of the homeobox antisense transcripts (HOXATs) with immune cell marker expression and tumor mutation burden (TMB). Heatmaps represent the association of the HOXATs expression with PD-L1 and CTLA4 in the pan-cancer **(A, B)**. For each pair, the top-right triangle represents the *p*-value, while the bottom-left triangle represents the correlation coefficient. *p < 0.05, **p < 0.01, ***p < 0.001. Heat map showing the correlation between the HOXAT expression and TMB **(C)**. The numbers on the heatmap represent the Spearman correlation values, where red and blue represent positive and negative correlations, respectively. *p < 0.05, **p < 0.01, ***p < 0.001.

The application of cancer immunotherapy has received significant attention in recent years. Here, we analyzed the relationship between the expression of HOXATs and 47 immune checkpoint genes in different immune infiltrated cells. Notably, the expression levels of most of the HOXATs showed a close relationship with the expression of immune markers in the various immune cells (Supplementary Figures S22–S39). Specifically, the expression levels of the majority of resting T cells, NK cells, eosinophils, and mast cells exhibited a significant correlation with the expression of HOXATs. This observation suggested that in some tumors, HOXATs possibly play a role in modulating the pattern of tumor immunity by regulating the expression levels of these immune checkpoint genes. Interestingly, HOXATs, including HOXB-AS1, HOXC-AS2, HAGLR, HOTAIR, and HOTAIRM1 showed significant positive correlation with the expression levels of immune checkpoint genes, including CTLA-4 and PD-L1, suggesting that these HOXATs are involved in the regulation of immune responses in different cancer types (Figures 4A,B). Therefore, these results confirmed the specific correlation between HOXAT expression levels and immune marker sets.

### Correlation Between HOXAT Expression and Tumor Mutation Burden in Pan-Cancer

TMB, which is considered an essential factor that affects the occurrence and progression of tumors, has been shown to affect response to cancer immunotherapy (Mao et al., 2018). Thus, we examined its association with HOXAT expression levels (Figure 4C). Interestingly, we observed that approximately half or more than half of the HOXATs were positively associated with TMB in cancers, including ACC, LGG, ESCA, HNSC, and MESO, but negatively correlated with TMB in SKCM. Notably, the higher expression levels of HOXATs in LGG showed their greater likelihood to bring about exposure of immune checkpoints, inducing a stronger anti-tumor immune response.

### Association Between HOXATs and Tumor Stemness in Pan-Cancers

**FIGURE 5:**
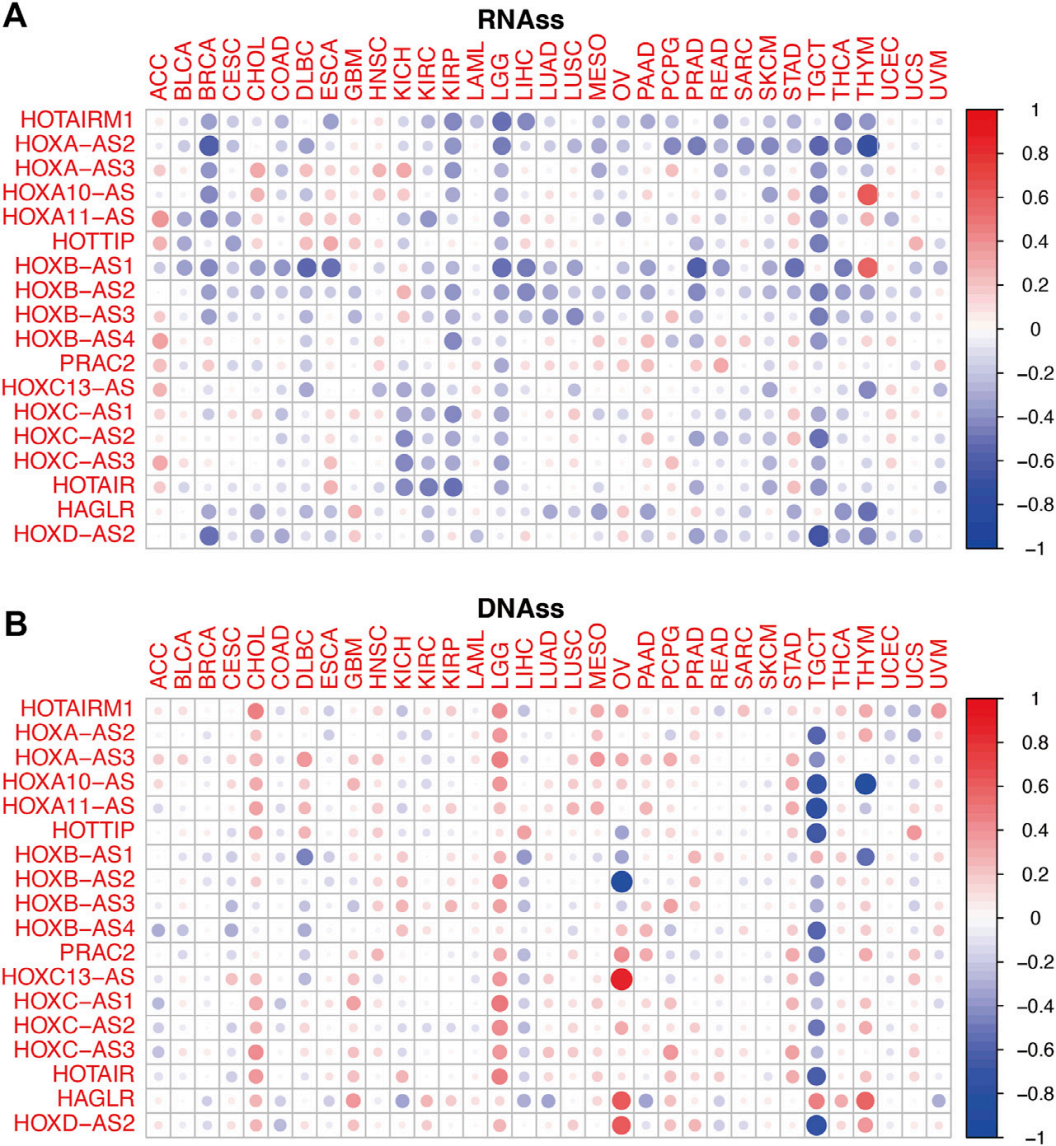
Relationship between the homeobox antisense transcripts (HOXATs) expression levels and the tumor stemness. Correlation matrix of the HOXATs expression and the cancer RNA and DNA stemness scores, RNAss **(A)** and DNAss **(B)**, respectively, based on Spearman correlation tests.

To conduct a preliminary study on the association between HOXATs and cancer stemness, tumor stemness based on RNA stemness scores (RNAss) and DNA stemness score (DNAss) were utilized (Figures 5A,B); lower RNAss or higher DNAss are associated with higher immune infiltration. Thus, we observed that HOXATs were negatively associated with RNAss in BRCA, KIRC, KIRP, LGG, READ, SKCM, TGCT, and THCA, and positively associated with DNAss in CHOL, GBM, LGG, and STAD. Notably, with the exception of HOTAIRM1 and HAGLR, all the other HOXATs showed negative association with RNAss and DNAss in TGCT.

### Potential Roles of HOXATs in Glioma Tumor Immunity and the TME

**FIGURE 6:**
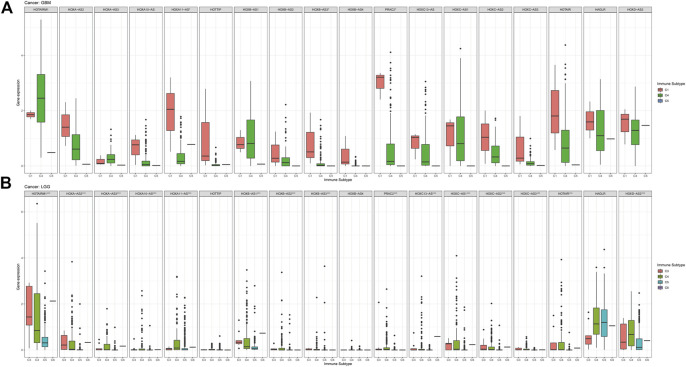
Association of the homeobox antisense transcripts (HOXATs) expression with the tumor immune microenvironment in glioblastoma (GBM) and low-grade glioma (LGG) patients based on TCGA. Association of the HOXATs expression with the immune infiltrate subtypes in GBM patients **(A)**. Association of HOXATs expression with immune infiltrate subtypes in LGG patients **(B)**. C1: wound healing; C2: INF-r dominant; C3: inflammatory; C4: lymphocyte depleted; C5: immunologically quiet; C6: TGFβ dominant,*p < 0.05, **p < 0.01, ***p < 0.001.

In this study, we also compared the expression levels of HOXAT genes across the immune subtypes in LGG and GBM based on the TCGA RNAseq database. First, the genes showed contrasting intra-tumor heterogeneities in the different immune subtypes of the different grades of glioma. Specifically, HOXB-AS3, HOXC13-AS, and HAGLR showed significant differences in expression level across C1 and C4 immune subtypes, indicating that HOXAT immune subtypes are minimally correlated with GBM (*p* < 0.05; Figure 6A). High HOXAT expression levels in GBM enhance immunosuppression and facilitate the escape of GBM from immune supervision. However, no significant association was observed in GBM in this regard. Similarly, our results were consistent with those of other studies, indicating that elevated expression levels of HOXATs, such as HOXA10-AS and HOXA-AS2, are associated with the malignancy of gliomas. Conversely, except for HOTTIP, HOXB-AS4, and HAGLR, the expression levels of the other 15 HOXATs were significantly different across the C1 and C4 immune subtypes in LGG, suggesting that higher HOXAT expression levels in LGG elicits a higher immune response, while a lower expression level induces immune escape (*p* < 0.001; Figure 6B).

**FIGURE 7:**
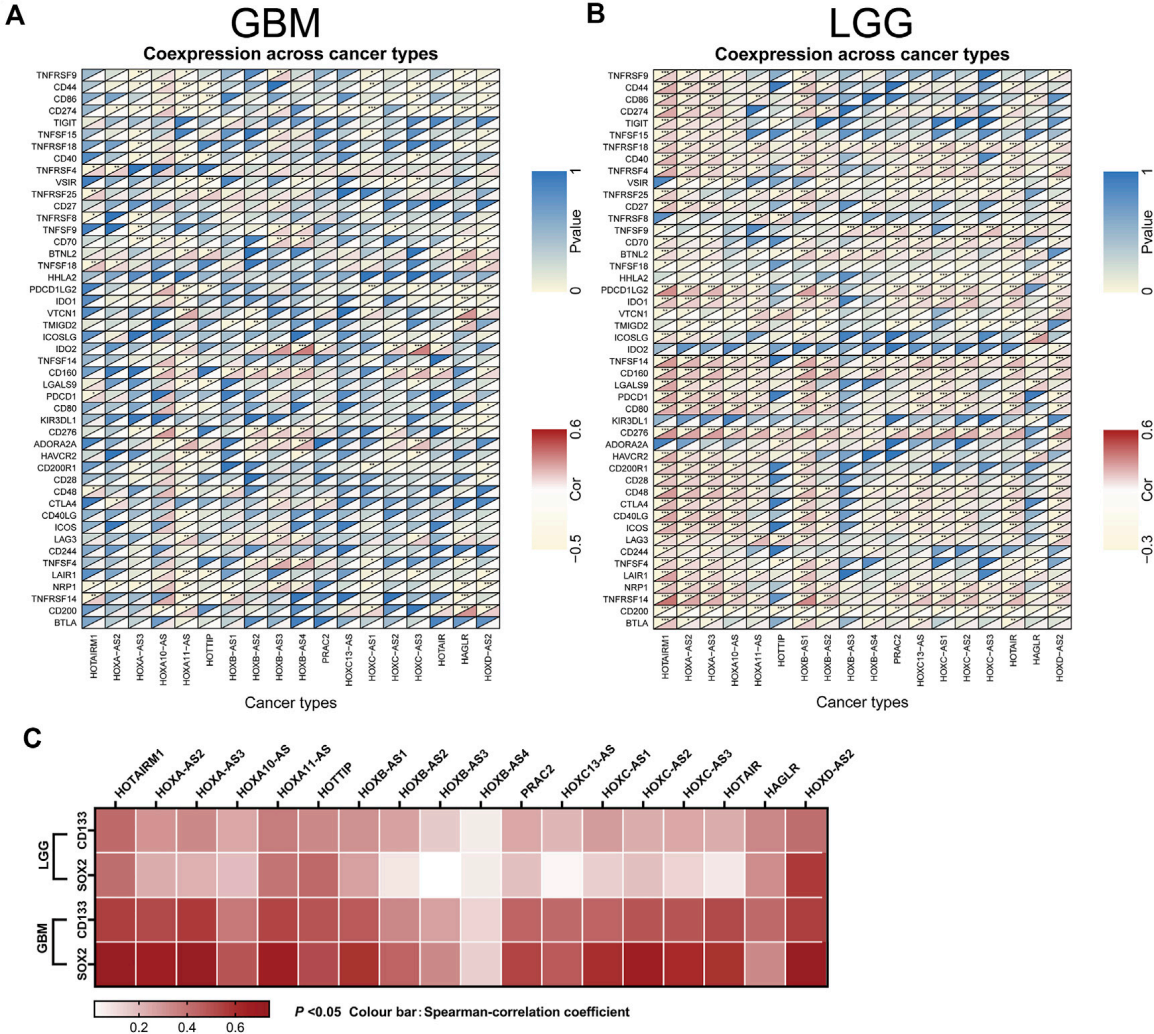
Association of the homeobox antisense transcripts (HOXATs) expression with stem cell and immune checkpoints in GBM and LGG. Co-expression analyses of the HOXATs and the immune checkpoints in the glioblastoma (GBM) **(A)** and low-grade glioma (LGG) patients **(B)**. For each pair, the top right triangle represents the *p*-value, while the bottom left triangle represents the correlation coefficient. GEPIA database showing HOXATs expression correlation with stem gene in high-grade glioma tissues compared with that in low-grade glioma tissues **(C)**. *p < 0.05, **p < 0.01, ***p < 0.001.

Next, an investigation of the correlation between immune checkpoint genes and the HOXATs in GBM and LGG showed the existence of a significant correlation between the majority of the immune checkpoint genes and the HOXATs in LGG compared with GBM (*p* < 0.001; Figures 7A,B), suggesting that HOXATs possibly regulate malignancy degree of gliomas by influencing the immune system. The correlation analysis between HOXATs and cancer stem cell-associated genes was investigated. It was found that HOXATs and CD133, SOX2 were differentially association within glioma tissues of different grades (Figure 7C). The advance of tumor grade, was related to the increasing of SOX2 and CD133 cells. To further verify the role of signaling activation in the aberrant expression of HOXATs in pan-cancer, functional annotation was performed using GSEA. Interestingly, based on Kyoto Encyclopedia of Genes and Genomes (KEGG) pathway analysis, except for HOXA-AS3 and HAGLR, 16 out of the 18 HOXATs were found to be enriched in several immune response pathways, including primary immunodeficiency, antigen processing and presentation, and the intestinal immune network corresponding to IGA.

### Critical Role of HOXB-AS1 in the Maintenance of the Properties of GSCs

**FIGURE 8:**
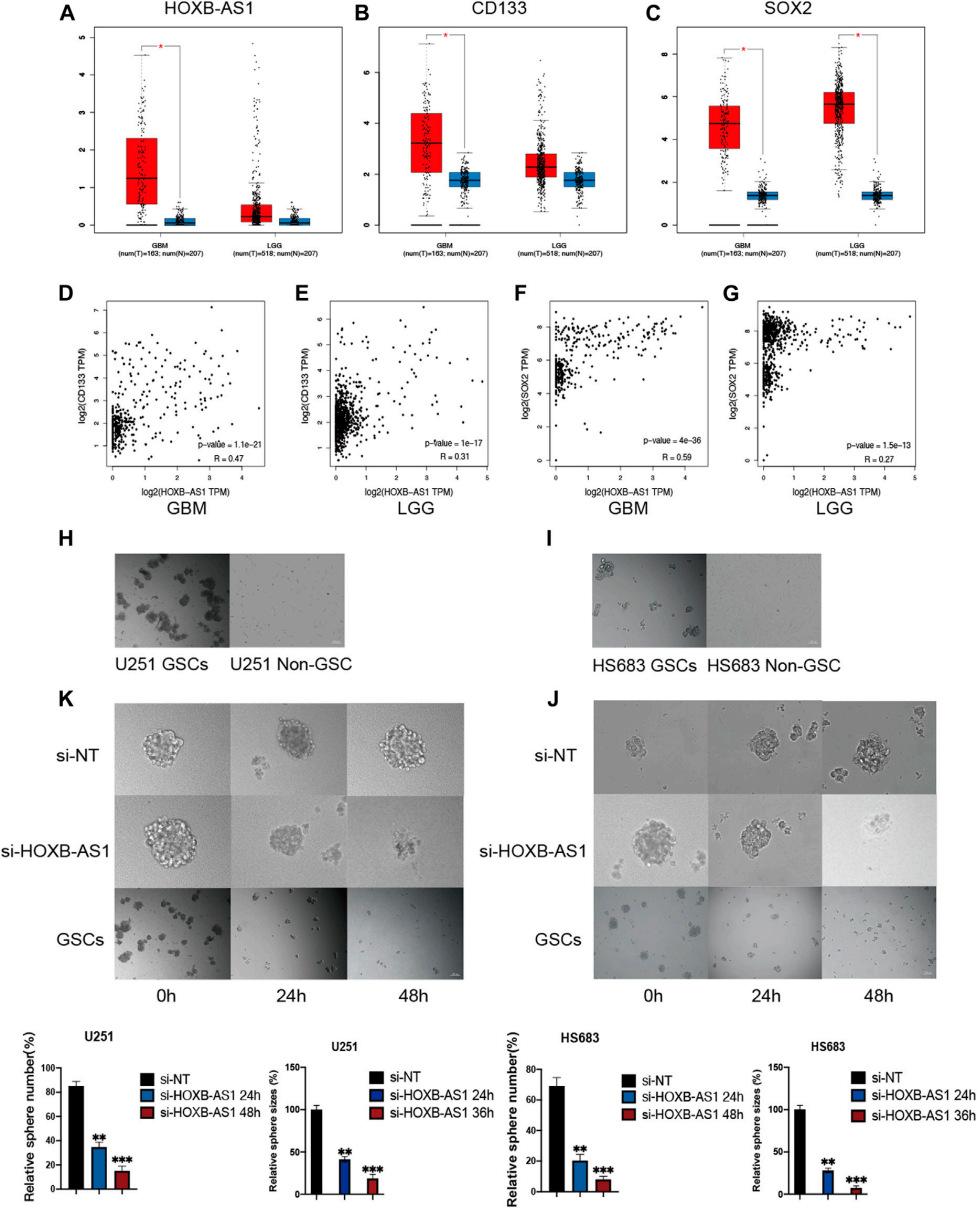
HOXB-AS1 is Required for GSC Maintenance and Survival. Comparison of HOXB-AS1, CD133 and SOX2 expression levels between LGG and GBM data sets **(A–C)**. The red column represents the glioma samples, and the blue column represents the normal samples. Correlations between differentially expressed HOXB-AS1 and stemness genes in GBM and LGG patients **(D–G)**. The representative images of neurosphere-cultured GSCs and 2 established GSC cells **(H, I)**. Silencing of HOXB-AS1 inhibits the proliferation and tumorigenicity of GSCs in U251and HS683. The effects of siRNA targeting of HOXB-AS1 on spheroid-forming ability in GBM and LGG cells were detected by sphere formation assay **(K, J)**. Bar: 100 μm *p < 0.05, **p < 0.01, ***p < 0.001.

To evaluate the association between HOXB-AS1 and the properties of GSCs in glioma, the expression level of HOXB-AS1 and the stemness markers that are associated with stemness in GBM and LGG were analyzed using the GEPIA online dataset (Figures 8A–C). As expected, HOXB-AS1 expression was significantly higher in GBM than in lower grade glioma tissues. Additionally, the stemness marker, CD133, which was significantly upregulated in GBM compared with normal tissues, could well distinguish between different grades of gliomas, while the other upregulated stemness marker, SOX2, was not different for the different grades of glioma. Moreover, correlation analysis demonstrated a strong correlation at transcriptional level between HOXB-AS1 and the stemness markers, CD133 and SOX2, especially in GBM (*p* < 0.001; Figures 8D–G). Further *in vitro* cell function experiments, which primarily involved cultured GBM and LGG cells in the normal culture medium with adherent culture conditions, confirmed that the established GSCs have a significantly higher neurosphere formation capacity than non-GSCs (*p* < 0.05; Figures 8H,I). Furthermore, sphere formation assay revealed that HOXB-AS1 knockdown inhibited spheroid formation in GSCs, indicating that HOXB-AS1 supports cancer stem cell growth. In conclusion, HOXB-AS1 was identified as being critical for the maintenance of the properties of GSCs (Figures 8J,K).

### Inhibition of Proliferation, Migration, and Invasion in Glioma Cells Following HOXB-AS1 Knockdown

**FIGURE 9:**
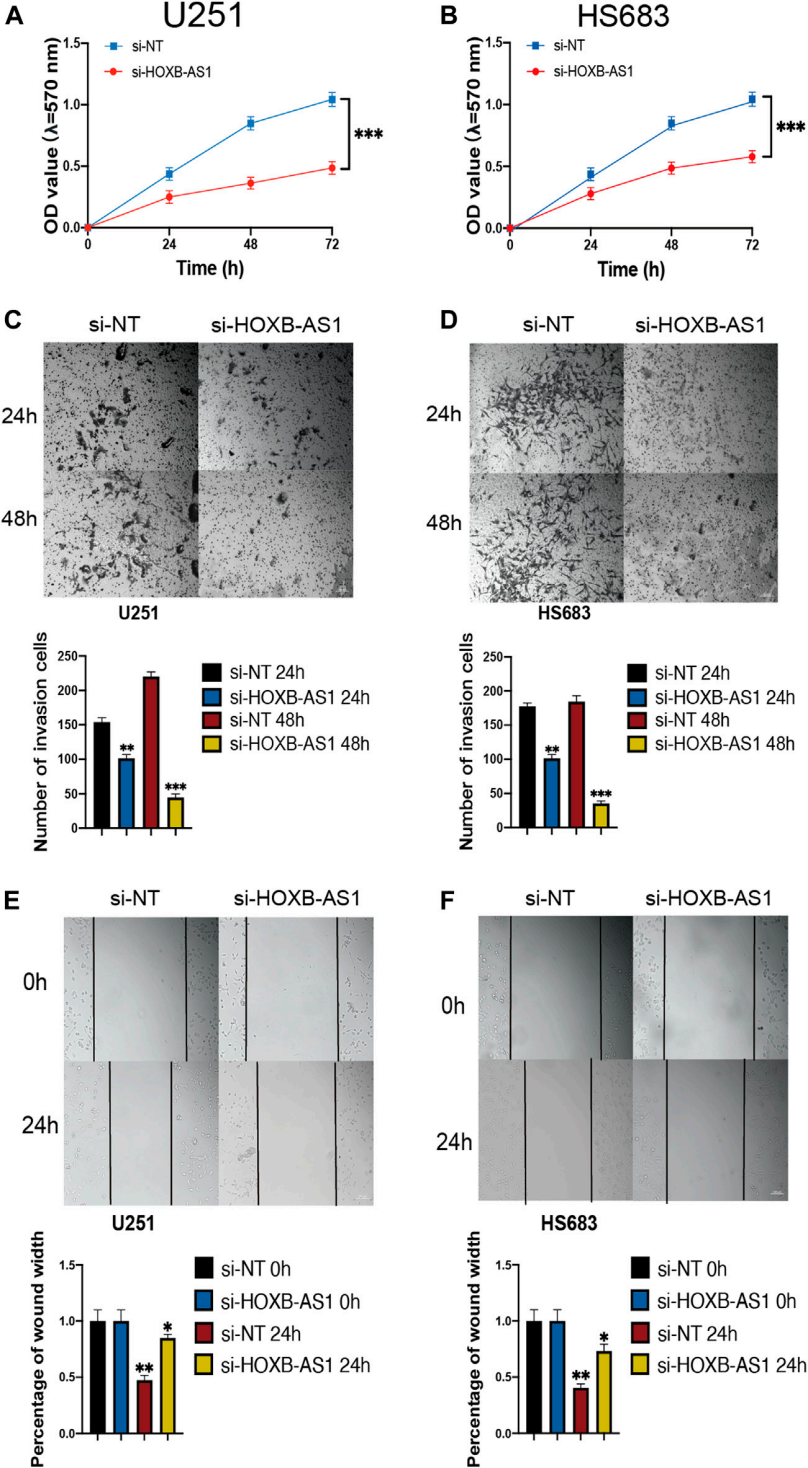
the homeobox antisense transcripts (HOXATs) enhanced the progression of glioma cells. Knockdown of HOXB-AS1 inhibited glioblastoma proliferation, metastasis, and invasion. Cell viability was measured by MTT assay in U251 and HS683 cell lines, and cell viability of cells with HOXB-AS1 knockdown was significantly inhibited **(A, B)**. Transwell migration assay: HOXB-AS1 knockdown significantly decreased the migration distance in U251and HS683 cells **(C, D)**. Invasion capacity was measured after HOXB-AS1 downregulation by transwell assay in U251 and HS683 cells **(E, F)**. Bar: 100 μm *p < 0.05, **p < 0.01, ***p < 0.001.

To further investigate the biological role of HOXB-AS1 in glioma cells, we depleted the expression of HOXB-AS1 using lncRNA Smart Silencer. As shown in Figures 9A,B, MTT assays were performed to detect cell viability. Thus, it was observed that U251 and HS683 cells transfected with HOXB-AS1 smart silencer exhibited a markedly increased rate of apoptosis compared with the control cells (*p* < 0.05). Further, transwell migration assay showed that HOXB-AS1 downregulation decreased U251 and HS683 migration (*p* < 0.05; Figures 9C,D), and similarly, wound healing assay indicated that HOXB-AS1 downregulation could significantly decreased invasion (*p* < 0.01; Figures 9E,F). These results indicated that HOXB-AS1 knockdown inhibited glioma cell proliferation, migration, and invasion.

## Discussion

Presently, it is well known that over 75% of the human genome is functional and encodes a large number of ncRNAs. A recent study provided strong evidence that lncRNAs play important roles in numerous biological processes, including chromatin modification, RNA processing, structural scaffold invasion modulation, and apoptosis (Zhou et al., 2017; Miyazato and Hayakawa, 2020). However, lncRNAs continue to be discovered and are yet to be functionally annotated. Therefore, studies with a focus on the elucidation of the biological functions of these lncRNAs and the identification of those that are disease-related are still required.

HOXATs play pivotal roles in tumor growth and metastatic spread (Meti et al., 2018). For instance, HOXB-AS1, which might function as a carcinogen and a promising therapeutic biomarker for the clinical treatment of patients with multiple myeloma, GBM, and endometrial carcinoma patients, has been shown to regulate cancer cell proliferation, migration, and invasion (Klebanov et al., 2019; Obaid et al., 2021). In this study, the expression levels of HOXATs in different cancer types were analyzed based on the TCGA database. The results of Cox regression analysis suggested that the high expression levels of half or more than half of the HOXATs was primarily associated with unfavorable outcomes in ACC, KIRC, LGG, and THYM. Additionally, certain HOXATs, including HOXA-AS2, HOXB-AS1, and HOTAIR possibly exerted distinct prognostic effects across the various cancer types (Quinn and Chang, 2016; Petermann et al., 2019).

Further, accumulating evidence also suggests that lncRNAs play important roles in immune regulation (Sharma et al., 2011; Sheng et al., 2018). For example, Li *et al.* identified immune lncRNAs as potential oncogenic biomarkers for pan-cancer characterization, based on the TCGA database (Sun et al., 2016). It has also been reported that the HOTAIR, influences the development of cancer invasion *via* immune responses (Wang et al., 2019a; Wang et al., 2019b). However, only a few examples have been identified thus far, this study represents the first comprehensive genome-wide analysis of HOXAT expression in pan-cancer, wherein the potential relationships between HOXATs and the TME in pan-cancers are explored *via* a bioinformatics analysis. Notably, HOXATs, such as HOTAIRM1, HOXA11-AS, HOXB-AS1, HOXB-AS4, HAGLR, and HOXC-AS1, showed significant correlation with immune cell infiltration and were likely to exhibit higher expression levels in immune cells, while the correlation between their expression levels and tumor dryness scores suggested that they possibly act primarily as tumor promoters in the TME. Additionally, as described above, we observed that HOXAT expression levels varied across the molecular subtypes within the same tumor type, and that the tumor suppression or promotion effect of the HOXATs may be subtype-specific.

The mechanisms of immune evasion by cancer cells are the targets of immunotherapies, and immune checkpoint inhibitors, including antibodies that block the programmed cell death of protein-1 (PD-1)/PD-L1 and the cytotoxic T lymphocyte associated protein 4 (CTLA-4), are receptors that attenuate T cell response. These have brought about changes in the strategies for the management of several cancers (Wang et al., 2015). Meanwhile, the co-expression of HOXATs in immune checkpoints and immune cells across 33 different cancer types indicated that the expression levels of HOXATs, such as HOXB-AS1, HOXC-AS2, HAGLR, HOTAIR, and HOTAIRM1 are significantly positively correlated with the expression of CTLA-4, PD-L1, and other immunocyte markers as well as immune cells, such as resting T cells, NK cells, eosinophils, and mast cells. Additionally, the identified signaling pathways showed association with immunity. Specifically, the results of this study indicated that these HOXATs may be important factors in the evolution of cancer development and might be involved in the regulation of immune response.

During cancer progression, tumor cells can progressively lose their differentiated phenotypes and acquire stem and progenitor-like features, which are believed to be responsible for tumor recurrence after standard chemotherapy and radiotherapy (Zhou et al., 2017). Recently, it has been hypothesized in several studies that cancer stem-like-cells, also known as tumor-initiating cells, mediate tumor initiation, progression, and metastasis (Wu et al., 2017). Interestingly, most of the HOXATs showed significant correlation with cancer stem cell-like features (DNAss and RNAss), suggesting that they likely play a role in tumor-initiating cells and are related to tumor resistance to drug treatment (Zhao et al., 2018a). HOXB-AS1 was identified as being critical for the maintenance of the properties of GSCs.

The immune microenvironment of GBM consists of anti-tumor immune response components (Yao et al., 2019; Yuan et al., 2020). Even though little is known regarding immunosuppression in this regard, a better understanding of the involvement and regulation of the immune system is essential for the treatment of glioblastoma. In this study, to observe the effects of HOXATs on the tumor immune microenvironment in gliomas, GBM and LGG were also compared in multiple ways, including immune subtype expression, immune markers, stromal scores, immune scores, and estimate scores using the ESTIMATE algorithm. The results obtained revealed that the expression levels of HOXATs are associated with diverse immune infiltration levels as well as tumor purity levels in gliomas, with high levels of intra-tumor heterogeneity in the immune infiltration corresponding to the different grades of the gliomas. Moreover, the HOXATs showed overexpression in the C4 immune subtype of GBM, i.e., the overexpression of HOXATs largely matched the immune profile of the C4 subtype, suggesting that HOXATs may play a negative role in the microenvironment, leading to an immunosuppressive TME, with poor prognosis, when compared with LGG. Further, the HOXATs also showed lower correlation with GBM than LGG in terms of both the immune and stromal scores, indicating that they are closely associated with the tumor immune microenvironment of LGG, but do not elicit a high immune response. Furthermore, the tumor immune microenvironment of GBM was altered when the glioma developed into GBM. Even though there was a high HOXAT expression that elicited an immune response, the HOXATs did not show any association with the TME. Therefore, we hypothesized that the early knockdown of HOXATs could inhibit the development of gliomas. HOXB-AS1 knockdown inhibited glioma cell proliferation, migration, and invasion.

With the continued exploration of lncRNAs (Zhang et al., 2018a)and the development of RNA-centric approaches (Zhang et al., 2018b), we will be able to identify the interaction targets of lncRNAs in a native context (Zhang et al., 2020). The identification of these targets will further improve our understanding of lncRNA-mediated pathway regulation and advance our understanding of lncRNA functions (Zhao et al., 2019). Simultaneously, the relationship between lncRNAs and TME represented an additional layer of tumor microenvironment complexity. Hence, it is important to clarify the roles of lncRNAs in TME. Additionally, the TME has a complex tissue-like structure with a rich phenotype as well as functional heterogeneity. Different concentrations of chemokines and cytokines, as well as interactions between the cells that make up the TME can activate epithelial-mesenchymal transition-related signaling pathways and control the generation of CSCs(Zhao et al., 2018b). In this study, we described the contribution of HOXATs in pan-cancer prognosis and TME modulation, demonstrating that HOXATs might serve as biomarkers for prognosis and immune response across cancers.

This study had some limitations. First, one of the major limitation of this study is that all of the experiments in this study were done in cultured cells. Second, the effects of the different HOXATs in the different cancer subtypes were not analyzed in detail, implying that further studies are needed in this regard to obtain more accurate results. These limitations notwithstanding, our results can significantly facilitate the clarification of the role of HOXATs in tumorigenesis, specifically with respect to immune response, the TME, and prognosis. This may be beneficial to researchers in this field with respect to the study of the roles of HOXATs and the development of personalized medicine for cancer treatment.

## Conclusion

This first comprehensive analysis of HOXATs expression in 33 different cancer types, analysed the role of the tumour microenvironment, and compared results in different grades of glioma. Therefore, we hypothesized that the early knockdown of HOXATs could stop the development of gliomas. We believe that the continued investigation of the HOXATs identified here will aid in the development of better immunotherapies for human cancer and other diseases.

## Data Availability

The original contributions presented in the study are included in the article/Supplementary Material, further inquiries can be directed to the corresponding authors.

## References

[B1] Akbari F. Peymani M. Salehzadeh A. Ghaedi K. (2020). Integrative In Silico and *In Vitro* Transcriptomics Analysis Revealed New lncRNAs Related to Intrinsic Apoptotic Genes in Colorectal Cancer. Cancer Cel Int 20, 546. 10.1186/s12935-020-01633-w PMC7653898 33292233

[B2] Bi Y. Mao Y. Su Z. Du J. Ye L. Xu F. (2020). HOXB‐AS1 Accelerates the Tumorigenesis of Glioblastoma via Modulation of HOBX2 and HOBX3 at Transcriptional and Posttranscriptional Levels. J. Cel Physiol 236, 93–106. 10.1002/jcp.29499 33459377

[B3] Carlsten M. Järås M. (2019). Natural Killer Cells in Myeloid Malignancies: Immune Surveillance, NK Cell Dysfunction, and Pharmacological Opportunities to Bolster the Endogenous NK Cells. Front. Immunol. 10, 2357. 10.3389/fimmu.2019.02357 31681270 PMC6797594

[B4] Chen Z. He A. Wang D. Liu Y. Huang W. (2016). Long Noncoding RNA HOTTIP as a Novel Predictor of Lymph Node Metastasis and Survival in Human Cancer: a Systematic Review and Meta-Analysis. Oncotarget 8, 14126–14132. 10.18632/oncotarget.12981 PMC5355167 27806342

[B5] Chen X. Li L. Q. Qiu X. Wu H. (2019). Long Non-Coding RNA HOXB-AS1 Promotes Proliferation, Migration and Invasion of Glioblastoma Cells via HOXB-AS1/miR-885-3p/HOXB2 axis. Neoplasma 66, 386–396. 10.4149/neo_2018_180606N377 30784279

[B6] Chen R. Zhang X. Wang C. (2019). LncRNA HOXB‐AS1 Promotes Cell Growth in Multiple Myeloma via FUT4 mRNA Stability by ELAVL1. J. Cel Biochem 121, 4043–4051. 10.1002/jcb.29573 31886581

[B7] Chen W. Li Q. Zhang G. Wang H. Zhu Z. Chen L. (2020). LncRNA HOXA‐AS3 Promotes the Malignancy of Glioblastoma through Regulating miR‐455‐5p/USP3 axis. J. Cel. Mol. Med. 24, 11755–11767. 10.1111/jcmm.15788 PMC7579690 32918360

[B8] Chi Y. Wang D. Wang J. Yu W. Yang J. (2019). Long Non-Coding RNA in the Pathogenesis of Cancers. Cells 8, 1015. 10.3390/cells8091015 PMC6770362 31480503

[B9] Cruz A. F. Fonseca N. A. Moura V. Simoes S. Moreira J. N. (2017). Targeting Cancer Stem Cells and Non-stem Cancer Cells: the Potential of Lipid-Based Nanoparticles. Curr. Pharm. Des. 23, 43. 10.2174/1381612823666171115105252 29141533

[B10] Dasti A. Cid-Samper F. Bechara E. Tartaglia G. G. (2020). RNA-centric Approaches to Study RNA-Protein Interactions *In Vitro* and In Silico. Methods 178, 11–18. 10.1016/j.ymeth.2019.09.011 31563541

[B11] Dong C. Y. Cui J. Li D. H. Li Q. Hong X. Y. (2018). HOXA10-AS: A Novel Oncogenic Long Non-Coding RNA in Glioma. Oncol. Rep. 40, 2573–2583. 10.3892/or.2018.6662 30132568 PMC6151881

[B12] Hao J. Yu J. (2018). Semaphorin 3C and its Receptors in Cancer and Cancer Stem-Like Cells. Biomedicines 6, 42. 10.3390/biomedicines6020042 PMC6027460 29642487

[B13] Klebanov N. Artomov M. Goggins W. B. Daly E. Daly M. J. Tsao H. (2019). Burden of Unique and Low Prevalence Somatic Mutations Correlates with Cancer Survival. Sci. Rep. 9, 4848. 10.1038/s41598-019-41015-5 30890735 PMC6425006

[B14] Li C. Liu S. Yan R. Han N. Wong K.-K. Li L. (2017). CD54-NOTCH1 axis Controls Tumor Initiation and Cancer Stem Cell Functions in Human Prostate Cancer. Theranostics 7, 67–80. 10.7150/thno.16752 28042317 PMC5196886

[B15] Li L. Wang Y. Song G. Zhang X. Gao S. Liu H. (2019). HOX Cluster-Embedded Antisense Long Non-coding RNAs in Lung Cancer. Cancer Lett. 450, 14–21. 10.1016/j.canlet.2019.02.036 30807784

[B16] Li K. Luo H. Huang L. Luo H. Zhu X. (2020). Microsatellite Instability: a Review of what the Oncologist Should Know. Cancer Cel Int 20, 16. 10.1186/s12935-019-1091-8 PMC6958913 31956294

[B17] Li Y. Jiang T. Zhou W. Li J. Li X. Wang Q. (2020). Pan-cancer Characterization of Immune-Related lncRNAs Identifies Potential Oncogenic Biomarkers. Nat. Commun. 11, 1000. 10.1038/s41467-020-14802-2 32081859 PMC7035327

[B18] Liang Y. Zhu H. Chen J. Lin W. Li B. Guo Y. (2020). Construction of Relapse-Related lncRNA-Mediated ceRNA Networks in Hodgkin Lymphoma. aoms 16, 1411–1418. 10.5114/aoms.2020.98839 PMC7667426 33224341

[B19] Lin S. Xu H. Zhang A. Ni Y. Xu Y. Meng T. (2020). Prognosis Analysis and Validation of m6A Signature and Tumor Immune Microenvironment in Glioma. Front. Oncol. 10, 541401. 10.3389/fonc.2020.541401 33123464 PMC7571468

[B20] Liu N. Wang Z. Liu D. Xie P. (2019). HOXC13-AS-miR-122-5p-SATB1-C-Myc Feedback Loop Promotes Migration, Invasion and EMT Process in Glioma. Onco. Targets Ther. 12, 7165–7173. 10.2147/OTT.S220027 31564901 PMC6731462

[B21] Liu D. Qiu M. Jiang L. Liu K. (2020). Long Noncoding RNA HOXB-AS1 Is Upregulated in Endometrial Carcinoma and Sponged miR-149-3p to Upregulate Wnt10b. Technol. Cancer Res. Treat. 19, 153303382096746. 10.1177/1533033820967462 PMC7592328 33073693

[B22] Lu C.-W. Zhou D.-D. Xie T. Hao J.-L. Pant O. P. Lu C.-B. (2018). HOXA11 Antisense Long Noncoding RNA (HOXA11-AS): A Promising lncRNA in Human Cancers. Cancer Med. 7 (8), 3792–3799. 10.1002/cam4.1571 29992790 PMC6089141

[B23] Mao C. Wang X. Liu Y. Wang M. Yan B. Jiang Y. (2018). A G3BP1-Interacting lncRNA Promotes Ferroptosis and Apoptosis in Cancer via Nuclear Sequestration of P53. Cancer Res. 78, 3484–3496. 10.1158/0008-5472.CAN-17-3454 29588351 PMC8073197

[B24] Meti N. Esfahani K. Johnson N. (2018). The Role of Immune Checkpoint Inhibitors in Classical Hodgkin Lymphoma. Cancers 10, 204. 10.3390/cancers10060204 PMC6025119 29914088

[B25] Miyazato K. Hayakawa Y. (2020). Pharmacological Targeting of Natural Killer Cells for Cancer Immunotherapy. Cancer Sci. 111 (6), 1869–1875. 10.1111/cas.14418 32301190 PMC7293096

[B26] Obaid M. Udden S. Alluri P. Mandal S. S. (2021). LncRNA HOTAIR Regulates Glucose Transporter Glut1 Expression and Glucose Uptake in Macrophages during Inflammation. Sci. Rep. 11, 232. 10.1038/s41598-020-80291-4 33420270 PMC7794310

[B27] Petermann F. Pękowska A. Johnson C. A. Jankovic D. Shih H. Y. Jiang K. (2019). The Magnitude of IFN-γ Responses Is Fine-Tuned by DNA Architecture and the Non-coding Transcript of Ifng-As1. Mol. Cel 75, 1229–e5. 10.1038/s41598-020-80291-410.1016/j.molcel.2019.06.025 PMC6754279 31377117

[B28] Quinn J. J. Chang H. Y. (2016). Unique Features of Long Non-coding RNA Biogenesis and Function. Nat. Rev. Genet. 17, 47–62. 10.1038/nrg.2015.10 26666209

[B29] Sharma S. Findlay G. M. Bandukwala H. S. Oberdoerffer S. Baust B. Li Z. (2011). Dephosphorylation of the Nuclear Factor of Activated T Cells (NFAT) Transcription Factor Is Regulated by an RNA-Protein Scaffold Complex. Proc. Natl. Acad. Sci. 108, 11381–11386. 10.1073/pnas.1019711108 21709260 PMC3136327

[B30] Sheng K. Lu J. Zhao H. (2018). ELK1-induced Upregulation of lncRNA HOXA10-AS Promotes Lung Adenocarcinoma Progression by Increasing Wnt/β-Catenin Signaling. Biochem. Biophysical Res. Commun. 501, 612–618. 10.1016/j.bbrc.2018.04.224 29729275

[B31] Sun T.-T. He J. Liang Q. Ren L.-L. Yan T.-T. Yu T.-C. (2016). LncRNA GClnc1 Promotes Gastric Carcinogenesis and May Act as a Modular Scaffold of WDR5 and KAT2A Complexes to Specify the Histone Modification Pattern. Cancer Discov. 6, 784–801. 10.1158/2159-8290.CD-15-0921 27147598

[B32] Wang Y. He L. Du Y. Zhu P. Huang G. Luo J. (2015). The Long Noncoding RNA lncTCF7 Promotes Self-Renewal of Human Liver Cancer Stem Cells through Activation of Wnt Signaling. Cell Stem Cell 16, 413–425. 10.1016/j.stem.2015.03.003 25842979

[B33] Wang L. Cho K. B. Li Y. Tao G. Xie Z. Guo B. (2019). Long Noncoding RNA (lncRNA)-Mediated Competing Endogenous RNA Networks Provide Novel Potential Biomarkers and Therapeutic Targets for Colorectal Cancer. Int. J. Mol. Sci. 20, 5758. 10.3390/ijms20225758 PMC6888455 31744051

[B34] Wang L. Wang L. Zhang X. (2019). Knockdown of lncRNA HOXA-AS2 Inhibits Viability, Migration and Invasion of Osteosarcoma Cells by miR-124-3p/E2F3. Onco Targets Ther. 12, 10851–10861. 10.2147/OTT.S220072 31853184 PMC6914662

[B35] Wu K. Zhao Z. Liu K. Zhang J. Li G. Wang L. (2017). Long Noncoding RNA Lnc-Sox5 Modulates CRC Tumorigenesis by Unbalancing Tumor Microenvironment. Cell Cycle 16, 1295–1301. 10.1080/15384101.2017.1317416 28632999 PMC5531622

[B36] Yao R.-W. Wang Y. Chen L.-L. (2019). Cellular Functions of Long Noncoding RNAs. Nat. Cel Biol 21, 542–551. 10.1038/s41556-019-0311-8 31048766

[B37] Yuan C. Ning Y. Pan Y. (2020). Emerging Roles of HOTAIR in Human Cancer. J. Cel Biochem 121, 3235–3247. 10.1002/jcb.29591 31943306

[B38] Zhang H. Liu Y. Yan L. Zhang M. Yu X. Du W. (2018). Increased Levels of the Long Noncoding RNA, HOXA-AS3, Promote Proliferation of A549 Cells. Cell Death Dis 9, 707. 10.1038/s41419-018-0725-4 29899328 PMC5999602

[B39] Zhang X. Chen W. Fan J. Wang S. Xian Z. Luan J. (2018). Disrupting CD47-Sirpα axis Alone or Combined with Autophagy Depletion for the Therapy of Glioblastoma. Carcinogenesis 39, 689–699. 10.1093/carcin/bgy041 29538621

[B40] Zhang X. Shao S. Li L. (2020). Characterization of Class-3 Semaphorin Receptors, Neuropilins and Plexins, as Therapeutic Targets in a Pan-Cancer Study. Cancers 12, 1816. 10.3390/cancers12071816 PMC7409005 32640719

[B41] Zhao X. Li X. Zhou L. Ni J. Yan W. Ma R. (2018). LncRNA HOXA11-AS Drives Cisplatin Resistance of Human LUAD Cells via Modulating miR-454-3p/Stat3. Cancer Sci. 109, 3068–3079. 10.1111/cas.13764 30099826 PMC6172072

[B42] Zhao W. Geng D. Li S. Chen Z. Sun M. (2018). LncRNA HOTAIR Influences Cell Growth, Migration, Invasion, and Apoptosis via the miR-20a-5p/HMGA2axis in Breast Cancer. Cancer Med. 7, 842–855. 10.1002/cam4.1353 29473328 PMC5852357

[B43] Zhao Q. Zhao S. Li J. Zhang H. Qian C. Wang H. (2019). TCF7L2 Activated HOXA-AS2 Decreased the Glucocorticoid Sensitivity in Acute Lymphoblastic Leukemia through Regulating HOXA3/EGFR/Ras/Raf/MEK/ERK Pathway. Biomed. Pharmacother. 109, 1640–1649. 10.1016/j.biopha.2018.10.046 30551418

[B44] Zhou M. Zhang Z. Zhao H. Bao S. Cheng L. Sun J. (2017). An Immune-Related Six-lncRNA Signature to Improve Prognosis Prediction of Glioblastoma Multiforme. Mol. Neurobiol. 55, 3684–3697. 10.1007/s12035-017-0572-9 28527107

