# Supplementary material for: A Pan-Cancer Landscape of HOX-Related lncRNAs and Their Association With Prognosis and Tumor Microenvironment
Source: Front Mol Biosci. 2021 Nov 5;8:767856. doi: 10.3389/fmolb.2021.767856 (PMC8602076; doi:10.3389/fmolb.2021.767856)
Supplement: Supplementary file 1 [file DataSheet1.ZIP › Supplementary_Material.docx]

Supplementary Material

**Supplementary Figure 1.** Expression levels of HOTAIRM1 in cancerous and adjacent normal tissues. Boxplot to show the distribution of HOTAIRM1 expression across all 33 cancer types.

**Supplementary Figure 2.** Expression levels of HOXA-AS2 in cancerous and adjacent normal tissues. Boxplot to show the distribution of HOXA-AS2 expression across all 33 cancer types.

**Supplementary Figure 3.** Expression levels of HOXA-AS3 in cancerous and adjacent normal tissues. Boxplot to show the distribution of HOXA-AS3 expression across all 33 cancer types.

**Supplementary Figure 4.** Expression levels of HOXA10-AS in cancerous and adjacent normal tissues. Boxplot to show the distribution of HOXA10-AS expression across all 33 cancer types.

**Supplementary Figure 5.** Expression levels of HOXA11-AS in cancerous and adjacent normal tissues. Boxplot to show the distribution of HOXA11-AS expression across all 33 cancer types.

**Supplementary Figure 6.** Expression levels of HOTTIP in cancerous and adjacent normal tissues. Boxplot to show the distribution of HOTTIP expression across all 33 cancer types.

**Supplementary Figure 7.** Expression levels of HOXB-AS1 in cancerous and adjacent normal tissues. Boxplot to show the distribution of HOXB-AS1 expression across all 33 cancer types.

**Supplementary Figure 8.** Expression levels of HOXB-AS2 in cancerous and adjacent normal tissues. Boxplot to show the distribution of HOXB-AS2 expression across all 33 cancer types.

**Supplementary Figure 9.** Expression levels of HOXB-AS3 in cancerous and adjacent normal tissues. Boxplot to show the distribution of HOXB-AS3 expression across all 33 cancer types.

**Supplementary Figure 10.** Expression levels of HOXB-AS4 in cancerous and adjacent normal tissues. Boxplot to show the distribution of HOXB-AS4 expression across all 33 cancer types.

**Supplementary Figure 11.** Expression levels of PRAC2 in cancerous and adjacent normal tissues. Boxplot to show the distribution of PRAC2 expression across all 33 cancer types.

**Supplementary Figure 12.** Expression levels of HOXC13-AS in cancerous and adjacent normal tissues. Boxplot to show the distribution of HOXC13-AS expression across all 33 cancer types.

**Supplementary Figure 13.** Expression levels of HOXC-AS1 in cancerous and adjacent normal tissues. Boxplot to show the distribution of HOXC-AS1 expression across all 33 cancer types.

**Supplementary Figure 14.** Expression levels of HOXC-AS2 in cancerous and adjacent normal tissues. Boxplot to show the distribution of HOXC-AS2 expression across all 33 cancer types.

**Supplementary Figure 15.** Expression levels of HOXC-AS3 in cancerous and adjacent normal tissues. Boxplot to show the distribution of HOXC-AS3 expression across all 33 cancer types.

**Supplementary Figure 16.** Expression levels of HOTAIR in cancerous and adjacent normal tissues. Boxplot to show the distribution of HOTAIR expression across all 33 cancer types.

**Supplementary Figure 17.** Expression levels of HAGLR in cancerous and adjacent normal tissues. Boxplot to show the distribution of HAGLR expression across all 33 cancer types.

**Supplementary Figure 18.** Expression levels of HOXD-AS2 in cancerous and adjacent normal tissues. Boxplot to show the distribution of HOXD-AS2 expression across all 33 cancer types.

**Supplementary Figure 19.** Meta-analyses of the relationship between OS and the expression of HOTTIP and HAGLR in different cancer patients, where high expression of HOTTIP (A) and HAGLR (B) led to a worse prognosis in pan-cancer patients.

**Supplementary Figure 20.** Kaplan Meier curve for the relationship between HOXATs expression in LGG with OS. 18 genes all associated with OS in LGG.

**Supplementary Figure 21.** Kaplan Meier curve for the relationship between HOXATs expression in LGG with DSS. 18 genes all associated with DSS in LGG.

**Supplementary Figure 22.** Correlation analysis between HOTAIRM1 expression and immune checkpoint genes and immune cell in pan-cancer. Heatmap representing the association of HOTAIRM1 expression with 47 immunocyte markers in pan-cancer (A). Heatmap representing the association of HOTAIRM1 expression with 21 kinds of immune cells in pan-cancer (B). For each immune checkpoint genes and immune cell pair, the triangles in the upper right corner are coloured to indicate the P value and the triangles in the lower left corner are coloured to indicate the correlation coefficient. *P < 0.05, **P < 0.01, ***P < 0.001.

**Supplementary Figure 23.** Correlation analysis between HOXA-AS2 expression and immune checkpoint genes and immune cell in pan-cancer. Heatmap representing the association of HOXA-AS2 expression with 47 immunocyte markers in pan-cancer (A). Heatmap representing the association of HOXA-AS2 expression with 21 kinds of immune cells in pan-cancer (B). For each immune checkpoint genes and immune cell pair, the triangles in the upper right corner are coloured to indicate the P value and the triangles in the lower left corner are coloured to indicate the correlation coefficient. *P < 0.05, **P < 0.01, ***P < 0.001.

**Supplementary Figure 24.** Correlation analysis between HOXA-AS3 expression and immune checkpoint genes and immune cell in pan-cancer. Heatmap representing the association of HOXA-AS3 expression with 47 immunocyte markers in pan-cancer (A). Heatmap representing the association of HOXA-AS3 expression with 21 kinds of immune cells in pan-cancer (B). For each immune checkpoint genes and immune cell pair, the triangles in the upper right corner are coloured to indicate the P value and the triangles in the lower left corner are coloured to indicate the correlation coefficient. *P < 0.05, **P < 0.01, ***P < 0.001.

**Supplementary Figure 25**. Correlation analysis between HOXA10-AS expression and immune checkpoint genes and immune cell in pan-cancer. Heatmap representing the association of HOXA10-AS expression with 47 immunocyte markers in pan-cancer (A). Heatmap representing the association of HOXA10-AS expression with 21 kinds of immune cells in pan-cancer (B). For each immune cell pair, the triangles in the upper right corner are coloured to indicate the P value and the triangles in the lower left corner are coloured to indicate the correlation coefficient. *P < 0.05, **P < 0.01, ***P < 0.001.

**Supplementary Figure 26**. Correlation analysis between HOXA11-AS expression and immune checkpoint genes and immune cell in pan-cancer. Heatmap representing the association of HOXA11-AS expression with 47 immunocyte markers in pan-cancer (A). Heatmap representing the association of HOXA11-AS expression with 21 kinds of immune cells in pan-cancer (B). For each immune cell pair, the triangles in the upper right corner are coloured to indicate the P value and the triangles in the lower left corner are coloured to indicate the correlation coefficient. *P < 0.05, **P < 0.01, ***P < 0.001.

**Supplementary Figure 27**. Correlation analysis between HOTTIP expression and immune checkpoint genes and immune cell in pan-cancer. Heatmap representing the association of HOTTIP expression with 47 immunocyte markers in pan-cancer (A). Heatmap representing the association of HOTTIP expression with 21 kinds of immune cells in pan-cancer (B). For each immune cell pair, the triangles in the upper right corner are coloured to indicate the P value and the triangles in the lower left corner are coloured to indicate the correlation coefficient. *P < 0.05, **P < 0.01, ***P < 0.001.

**Supplementary Figure 28**. Correlation analysis between HOXB-AS1 expression and immune checkpoint genes and immune cell in pan-cancer. Heatmap representing the association of HOXB-AS1 expression with 47 immunocyte markers in pan-cancer (A). Heatmap representing the association of HOXB-AS1 expression with 21 kinds of immune cells in pan-cancer (B). For each immune cell pair, the triangles in the upper right corner are coloured to indicate the P value and the triangles in the lower left corner are coloured to indicate the correlation coefficient. *P < 0.05, **P < 0.01, ***P < 0.001.

**Supplementary Figure 29**. Correlation analysis between HOXB-AS2 expression and immune checkpoint genes and immune cell in pan-cancer. Heatmap representing the association of HOXB-AS2 expression with 47 immunocyte markers in pan-cancer (A). Heatmap representing the association of HOXB-AS2 expression with 21 kinds of immune cells in pan-cancer (B). For each immune cell pair, the triangles in the upper right corner are coloured to indicate the P value and the triangles in the lower left corner are coloured to indicate the correlation coefficient. *P < 0.05, **P < 0.01, ***P < 0.001.

.

**Supplementary Figure 30**. Correlation analysis between HOXB-AS3 expression and immune checkpoint genes and immune cell in pan-cancer. Heatmap representing the association of HOXB-AS3 expression with 47 immunocyte markers in pan-cancer (A). Heatmap representing the association of HOXB-AS3 expression with 21 kinds of immune cells in pan-cancer (B). For each immune cell pair, the triangles in the upper right corner are coloured to indicate the P value and the triangles in the lower left corner are coloured to indicate the correlation coefficient. *P < 0.05, **P < 0.01, ***P < 0.001.

**Supplementary Figure 31**. Correlation analysis between HOXB-AS4 expression and immune checkpoint genes and immune cell in pan-cancer. Heatmap representing the association of HOXB-AS4 expression with 47 immunocyte markers in pan-cancer (A). Heatmap representing the association of HOXB-AS4 expression with 21 kinds of immune cells in pan-cancer (B). For each immune cell pair, the triangles in the upper right corner are coloured to indicate the P value and the triangles in the lower left corner are coloured to indicate the correlation coefficient. *P < 0.05, **P < 0.01, ***P < 0.001.

**Supplementary Figure 32**. Correlation analysis between PRAC2 expression and immune checkpoint genes and immune cell in pan-cancer. Heatmap representing the association of PRAC2 expression with 47 immunocyte markers in pan-cancer (A). Heatmap representing the association of PRAC2 expression with 21 kinds of immune cells in pan-cancer (B). For each immune cell pair, the triangles in the upper right corner are coloured to indicate the P value and the triangles in the lower left corner are coloured to indicate the correlation coefficient. *P < 0.05, **P < 0.01, ***P < 0.001.

**Supplementary Figure 33**. Correlation analysis between HOXC13-AS expression and immune checkpoint genes and immune cell in pan-cancer. Heatmap representing the association of HOXC13-AS expression with 47 immunocyte markers in pan-cancer (A). Heatmap representing the association of HOXC13-AS expression with 21 kinds of immune cells in pan-cancer (B). For each immune cell pair, the triangles in the upper right corner are coloured to indicate the P value and the triangles in the lower left corner are coloured to indicate the correlation coefficient. *P < 0.05, **P < 0.01, ***P < 0.001.

**Supplementary Figure 34**. Correlation analysis between HOXC-AS1 expression and immune checkpoint genes and immune cell in pan-cancer. Heatmap representing the association of HOXC-AS1 expression with 47 immunocyte markers in pan-cancer (A). Heatmap representing the association of HOXC-AS1 expression with 21 kinds of immune cells in pan-cancer (B). For each immune cell pair, the triangles in the upper right corner are coloured to indicate the P value and the triangles in the lower left corner are coloured to indicate the correlation coefficient. *P < 0.05, **P < 0.01, ***P < 0.001.

**Supplementary Figure 35.** Correlation analysis between HOXC-AS2 expression and immune checkpoint genes and immune cell in pan-cancer. Heatmap representing the association of HOXC-AS2 expression with 47 immunocyte markers in pan-cancer (A). Heatmap representing the association of HOXC-AS2 expression with 21 kinds of immune cells in pan-cancer (B). For each immune cell pair, the triangles in the upper right corner are coloured to indicate the P value and the triangles in the lower left corner are coloured to indicate the correlation coefficient. *P < 0.05, **P < 0.01, ***P < 0.001.

**Supplementary Figure 36.** Correlation analysis between HOXC-AS3 expression and immune checkpoint genes and immune cell in pan-cancer. Heatmap representing the association of HOXC-AS3 expression with 47 immunocyte markers in pan-cancer (A). Heatmap representing the association of HOXC-AS3 expression with 21 kinds of immune cells in pan-cancer (B). For each immune cell pair, the triangles in the upper right corner are coloured to indicate the P value and the triangles in the lower left corner are coloured to indicate the correlation coefficient. *P < 0.05, **P < 0.01, ***P < 0.001.

**Supplementary Figure 37.** Correlation analysis between HOTAIR expression and immune checkpoint genes and immune cell in pan-cancer. Heatmap representing the association of HOTAIR expression with 47 immunocyte markers in pan-cancer (A). Heatmap representing the association of HOTAIR expression with 21 kinds of immune cells in pan-cancer (B). For each immune cell pair, the triangles in the upper right corner are coloured to indicate the P value and the triangles in the lower left corner are coloured to indicate the correlation coefficient. *P < 0.05, **P < 0.01, ***P < 0.001.

**Supplementary Figure 38.** Correlation analysis between HAGLR expression and immune checkpoint genes and immune cell in pan-cancer. Heatmap representing the association of HAGLR expression with 47 immunocyte markers in pan-cancer (A). Heatmap representing the association of HAGLR expression with 21 kinds of immune cells in pan-cancer (B). For each immune cell pair, the triangles in the upper right corner are coloured to indicate the P value and the triangles in the lower left corner are coloured to indicate the correlation coefficient. *P < 0.05, **P < 0.01, ***P < 0.001.

**Supplementary Figure 39.** Correlation analysis between HOXD-AS2 expression and immune checkpoint genes and immune cell in pan-cancer. Heatmap representing the association of HOXD-AS2 expression with 47 immunocyte markers in pan-cancer (A). Heatmap representing the association of HOXD-AS2 expression with 21 kinds of immune cells in pan-cancer (B). For each immune cell pair, the triangles in the upper right corner are coloured to indicate the P value and the triangles in the lower left corner are coloured to indicate the correlation coefficient. *P < 0.05, **P < 0.01, ***P < 0.001.

**Supplementary Figure 40.** Gene set enrichment analysis (GSEA) of the biological pathways and processes, which were found to be correlated with the immune score values in TCGA cohort.
